# Fluorescence Efficiency of Laser Dyes*

**DOI:** 10.6028/jres.080A.044

**Published:** 1976-06-01

**Authors:** K. H. Drexhage

**Affiliations:** Research Laboratories, Eastman Kodak Company, Rochester, New York 14650

**Keywords:** Aminocoumarins, carbazine dyes, deuterium effect, fluorescence quantum yield, laser dyes, molecular structure, oxazine dyes, quenching, xanthene dyes

## Abstract

The fluorescence efficiency of xanthene dyes, oxazine dyes, and 7-aminocoumarins is discussed. Relations with the molecular structure are pointed out and dependence on solvent and temperature is explained. Several new fluorescence standards are suggested.

## 1. Introduction

The recent development of the dye laser [1]^1^ has opened up an important new field of applications for organic dyes. This has led to a renewed interest in the theory of nonradiative transitions in dyes and also to the synthesis of new highly fluorescent dyes. In this article we review briefly the relations between fluorescence and molecular structure in the most important classes of laser dyes: xanthenes, oxazines, and 7-aminocoumarins. Following this discussion specific suggestions for improved fluorescence standards are made.^2^

## 2. Xanthene Dyes

The chromophore of xanthene dyes has typically the following structures

**Figure:**
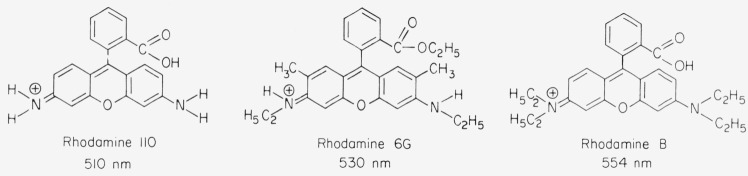

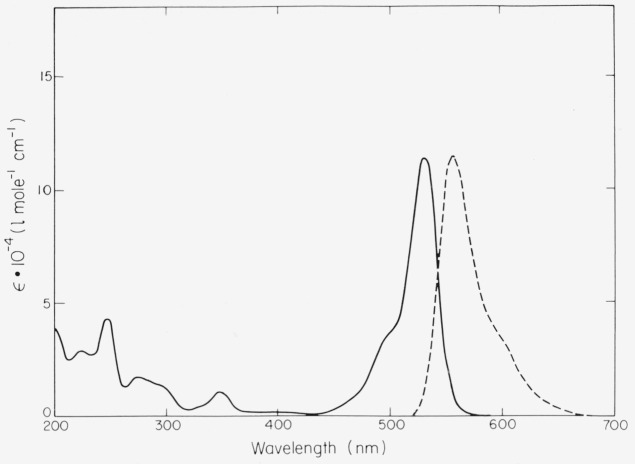


**Figure 1:**
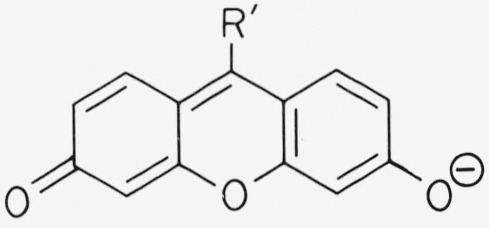
Molecular structure of rhodamine dyes; absorption maximum in ethanol.

Depending on the end groups, in particular number and type of the substituents *R*, the maximum of the main absorption band falls somewhere in the range 480–580 nm. The transition moment is parallel to the long axis of the molecule [2]. With *R′ = H* and amino end groups the dyes are called pyronins. Due to a convenient syntheses with phthalic anhydride many xanthene dyes have *R′* = carboxyphenyl and are called fluorescein or rhodamines (fig. 1). The methyl substituents of Rhodamine 6G have practically no influence on the optical properties of the dye except for the dimerization in aqueous solution which we are not concerned with here. The absorption maximum of the main band occurs almost at the same wavelength in pyronins and rhodamines, if the end groups are identical. The absorption band near 350 nm is stronger in rhodamines than in pyronins, and rhodamines show a slightly larger Stokes shift than pyronins. The chemical stability of rhodamines is generally superior, as pyronins in alkaline solution are readily oxidized by dissolved oxygen to form a colorless, blue fluorescing xanthone.

Rhodamines like Rhodamine B react to pH variations in an interesting manner. The carboxyl group is completely protonated in acidic solution, but dissociates in alkaline solution. The negative charge has an inductive effect on the central carbon atom of the chromophore. The maxima of the main absorption band and of the fluorescence are shifted to shorter wavelengths, and the extinction coefficient at the absorption maximum is slightly reduced. While these effects are rather weak in aqueous solution [3], the wavelength shift amounts to about 10 nm in polar organic solvents (methanol, ethanol, etc.) [4, 5, 6, 7]. When Rhodamine B, which is usually obtained as the hydrochloride,

**Figure:**
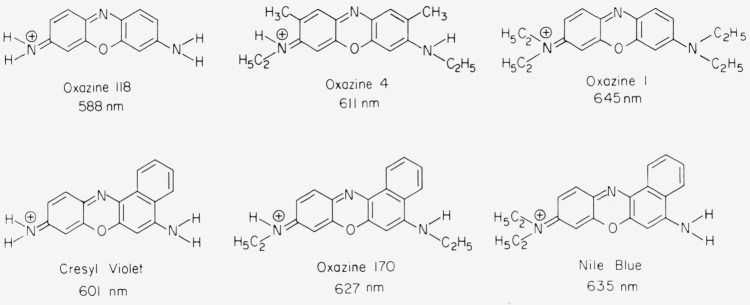


is dissolved in ethanol, the relative amounts of the two forms of the dye are determined by this acid-base equilibrium. The dissociation increases on dilution causing a shift of absorption and fluorescence to shorter wavelengths. This concentration dependence of the spectra is therefore not due to a monomerdimer equilibrium, as was claimed in recent papers [8, 9]. In less polar organic solvents (e.g., acetone) the unprotonated (zwitterionic) dye undergoes a reversible conversion to a lactone:

**Figure:**
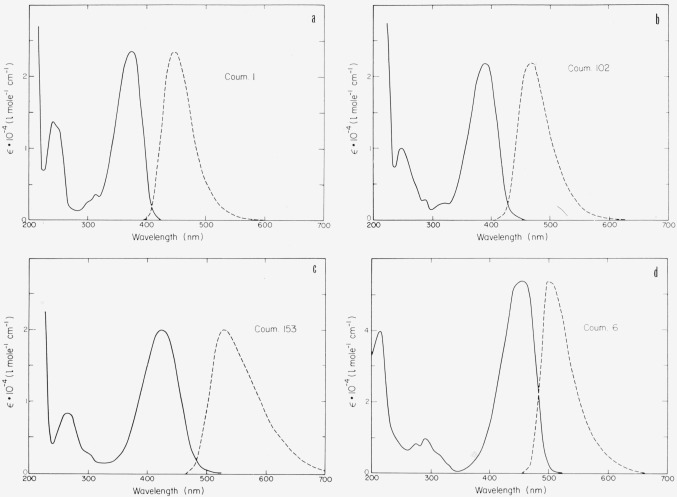


Because the conjugation of the chromophore is interrupted, this is a colorless compound. On addition of acid or complex-forming metal ions the chromophore is regenerated. If the carboxyl group is esterified as in Rhodamine 6G, all these reactions do not take place, and the absorption maximum is independent of pH and concentration (except for aqueous solutions, where aggregation takes place, and for strongly acidic conditions, where protonation of the amino groups occurs).

**Figure 2:**
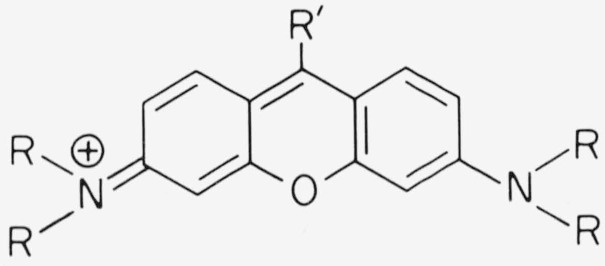
Rhodamine 6G in ethanol. Absorption spectrum (ϵ molar decadic extinction coefficient); quantum spectrum of fluorescence (arbitrary units).

Because of the symmetry of the rhodamine chromophore, there is no dipole moment parallel to its long axis. As a consequence, the Stokes shift is small, and the fluorescence band overlaps strongly the absorption band (fig. 2) [10]. Also, there is only little variation of absorption and fluorescence maxima with solvent polarity [4, 5]. Provided there are no heavy-atom substituents, the rate of intersystem crossing from *S*_1_ is very low in xanthene dyes, which is in accordance with our loop rule [11]. Phosphorescence is weak even in low temperature glasses and has a lifetime in the order of 0.1 s, which is short compared with, for instance, acriflavine and some aromatic compounds.

The fluorescence of rhodamines is quenched externally by I^−^ and SCN^−^, less efficiently by Br^−^ and Cl^−^. No quenching was observed by ClO_4_^−^ and BF_4_^−^ [4, 5]. The quenching process apparently involves a charge transfer from the anion to the excited dye molecule. It is not a heavy-atom effect. In polar solvents like ethanol, Rhodamine 6G-iodide is completely dissociated at a concentration of 10^−4^ mol/l and no quenching takes place, because the lifetime of the excited state is of the order of a few nanoseconds, much shorter than the diffusion time the quencher would need to reach an excited dye molecule. In less polar solvents like chloroform the dye salt does not dissociate and its fluorescence is totally quenched. The perchlorate of Rhodamine 6G behaves strikingly differently: its fluorescence efficiency is independent [12] of solvent polarity (0.95 based on the value 0.90 for fluorescein). It is, however, reduced by heavy-atom solvents (0.40 in iodomethane), and the fluorescence of this dye is completely quenched by nitrobenzene, presumably due to the high electron affinity of this solvent.

There are two structural features that influence the rate of internal conversion in xanthene dyes: mobility of the amino groups and hydrogen vibrations. We found that the fluorescence efficiency of rhodamines that carry two alkyl substituents at each nitrogen, e.g., Rhodamine B, varies strongly with solvent and temperature [4, 5, 11]. We ascribe this to mobility of the amino groups. If the amino groups are rigidized as in the new dye Rhodamine 101 (λ_abs_ = 577 nm in ethanol) the quantum efficiency is practically unity,

**Figure:**
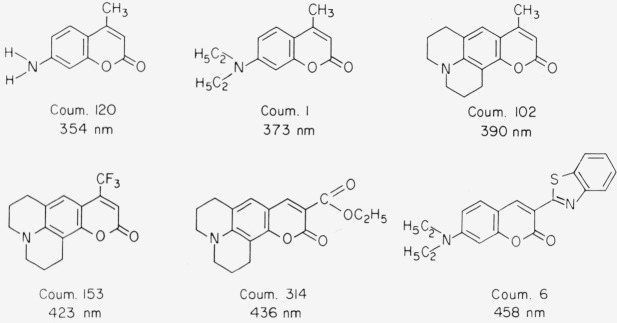


independent of solvent and temperature. It is interesting that the amino groups are not mobile when they are less than fully alkylated (Rhodamine 6G, Rhodamine 110). However, in such dyes there is a probability

**Figure:**
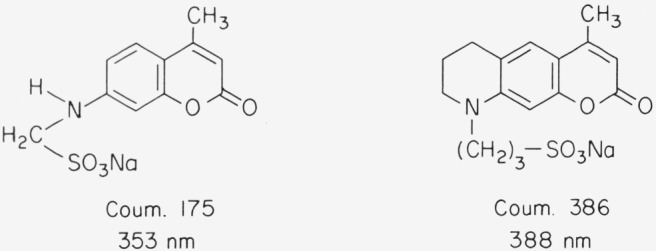


that the electronic excitation energy is funneled into *N-H* vibrations, causing nonradiative decay to *S*_0_. Although this effect is rather weak in xanthene dyes, it is noticeable for instance in Rhodamine 6G. On solution in *O*-deuterated ethanol, *H* is exchanged for *D* in the amino groups of the dye. With the greater mass of deuterium the nonradiative decay is less likely and the fluorescence efficiency increases from 0.95 to 0.99.

## 3. Oxazine Dyes

**Figure 3:**
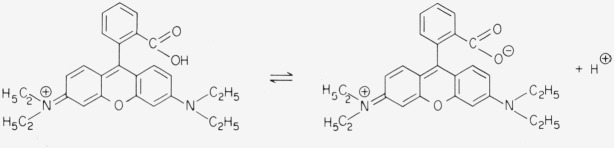
Molecular structure of oxazine dyes; absorption maximum in ethanol.

When the central carbon atom in the xanthene chromophore is replaced by nitrogen, the chromophore of an oxazine dye is obtained. The central *N*-atom acts as a sink for the *π*-electrons, causing a wavelength shift of about 80 nm to the red. As within the xanthene class the absorption of oxazines also shifts to the red with increasing alkylation of the amino groups (fig. 3). Apart from the pronounced red shift of the *S*_0_*–S*_1_ transition there is little difference between the electronic transitions of oxazines and rhodamines [2]. The electron-withdrawing effect of the central *N*-atom causes the amino groups to be more acidic. Thus addition of a little base to a solution of Oxazine 4 in ethanol changes the color from blue to red due to deprotonation of one of the amino groups:

Oxazines with fully alkylated amino groups do not undergo any change on addition of base. On the other hand, all oxazines are protonated in strong acids.

Some oxazine dyes have a structure modified by an added benzo group (fig. 3). This causes a slight red shift of absorption and fluorescence. Furthermore, the shape of the absorption spectrum is different than in other oxazine dyes and depends on the temperature [13]. These effects are probably caused by steric interference of the amino group with a hydrogen of the added benzo group [11].

The triplet yield of oxazines is generally very low in accordance with the loop rule. No phosphorescence has been observed in these dyes. The fluorescence quenching processes, discussed for xanthene dyes, are also found with oxazines. Owing to the smaller energy difference between *S*_1_ and *S*_0_, internal conversion plays a greater role in oxazines than in rhodamines. Thus the fluorescence efficiency of Oxazine l-perchlorate is less than 0.1 in ethanol, but much higher in dichloromethane and in 1,2-dichlorobenzene. Likewise, the effect of hydrogen vibrations is much more pronounced. The fluorescence efficiency of Oxazine 4 is a factor of 2 higher in *O*-deuterated ethanol than in normal ethanol.

## 4. 7-Aminocoumarins

The most important laser dyes in the blue and green region of the spectrum are coumarin derivatives that have an amino group in 7-position:

**Figure:**
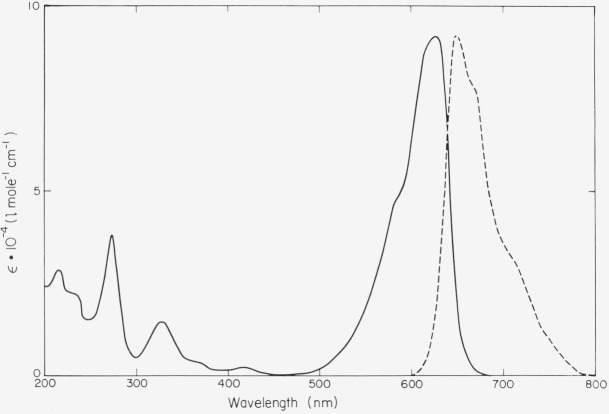


**Figure 4a, b, c, and d:**
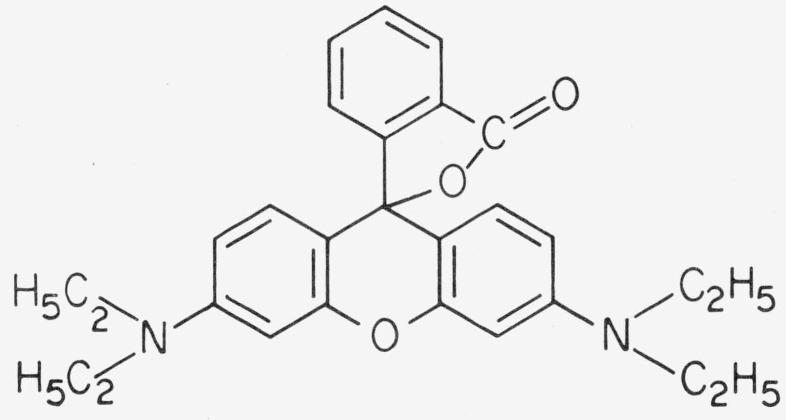
7-Aminocoumarins in ethanol. Absorption spectrum (ϵ molar decadic extinction coefficient); quantum spectrum of fluorescence (arbitrary units).

**Figure 5:**
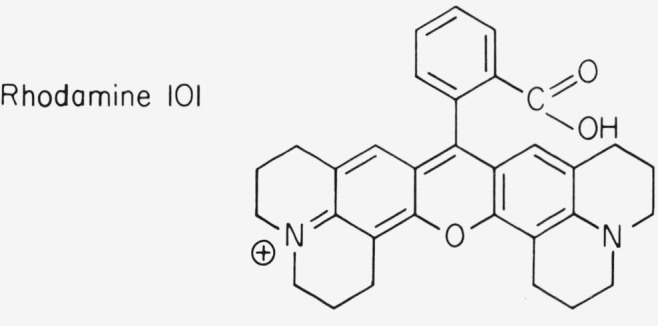
Molecular structure of 7-aminocoumarin derivatives; absorption maximum in ethanol.

**Figure 6:**
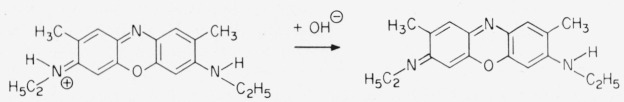
Molecular structure of water-soluble 7-aminocoumarins; absorption maximum in water.

The chromophore is not symmetric as in the xanthenes and oxazines discussed above. The ground state can be described by structure A, the excited state *S*_1_ by B. While there is some dipole moment in the ground state, it is much greater in the excited state. As a consequence these compounds show a large Stokes shift, in particular in polar solvents (fig. 4). Absorption and fluorescence maxima depend much more on solvent polarity than is the case with xanthenes and oxazines [4, 5]. A large number of highly fluorescent derivatives have been synthesized in recent years [4, 5, 14–18]. Here we can give only a few examples (fig. 5). Absorption and fluorescence of 7-aminocoumarins are not influenced by a small amount of base present in the solution. However, they react with strong acids in a variety of ways [4, 5, 11]. The amino group of Coumarin 1, for instance, is easily protonated and thus decoupled from the chromophore. As a consequence the absorption band at 373 nm disappears. This reaction is inhibited, if the amino group is rigidized as in Coumarin 102. In the excited state *S*_1_, the keto group is more basic than the amino group and thus is protonated if sufficient acid is present so that the diffusion is faster than the decay of *S*_1_. Frequently a new fluorescence band appears due to the protonated form. Most coumarins are very soluble in organic solvents, but insoluble in water. However, derivatives have been reported recently that are highly soluble in water (fig. 6) [18].

## 5. Fluorescence Standards

The optical properties of an ideal fluorescence standard should be independent of the environment (solvent composition, temperature, etc.). This means, among other things, that the compound should not be involved in chemical equilibria. Alkaline solutions are to be avoided, because their alkalinity changes gradually due to absorption of carbon dioxide. Furthermore the fluorescence should not be quenched by oxygen and the fluorescence efficiency should be independent of temperature variations. The commonly used fluorescence standards fail badly on one or more of these requirements. A number of better standards can be suggested on the basis of the foregoing discussions.

### Coumarin 1

This compound (7-diethylamino-4-methylcoumarin) is the most readily available 7-aminocoumarin. It is easily purified by recrystallization from ethanol. It is highly soluble in most organic solvents, but not in water. The fluorescence quantum efficiency is about 0.5 in ethanol, generally higher in less polar solvents, and is almost independent of temperature. There is little or no quenching by oxygen. A disadvantage is the possible protonation in acidic solvents. If this is a problem, Coumarin 102 or Coumarin 153 may be used. The latter compound is particularly interesting because of its large Stokes-shift and the very broad fluorescence spectrum. The fluorescence efficiency of Coumarin 102 was measured as 0.6 and that of Coumarin 153 as 0.4 (both in ethanol). Another compound, possibly useful as a standard, is Coumarin 6 (quantum efficiency 0.8 in ethanol), which absorbs at longer wavelengths than most other coumarin derivatives. The latter compounds are commercially available, but the price is still high. However, this should not be a deterrent, as the price will certainly come down and generally only a few milligrams are required. We feel that Coumarin 1 is superior to quinine bisulfate in every respect. It can be used in almost any solvent except water.

### Rhodamine 6G-ClO_4_

As was pointed out previously in this article, the frequently used standard Rhodamine B undergoes acid-base reactions that affect its optical properties. Its fluorescence efficiency depends strongly on type of solvent and temperature. Therefore it is not surprising that the quantum yield values reported in the literature vary considerably. As discussed above, Rhodamine 6G-perchlorate is superior to Rhodamine B, because its quantum efficiency has the value 0.95 almost independent of solvent and temperature. The dye chloride is readily available in rather pure form. It can be further purified by column chromatography on basic alumina with ethanol or methanol as the solvent. The perchlorate is insoluble in water and is easily prepared by adding HClO_4_ to an aqueous solution of the dye chloride. It can be recrystallized from alcohol-water mixtures. The ethyl ester perchlorate of Rhodamine 101 has the same useful properties as Rhodamine 6G-perchlorate, while absorption and fluorescence are shifted about 50 nm to the red. However, this dye is not yet commercially available.

### Oxazine 170-ClO_4_

**Figure 7:**
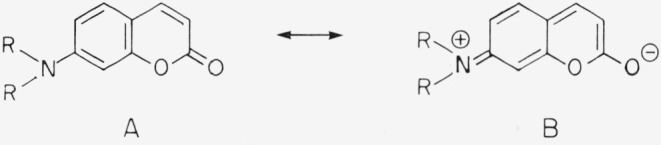
Oxazine 170 in ethanol. Absorption spectrum (ϵ molar decadic extinction coefficient); quantum spectrum of fluorescence (arbitrary units).

Of all available oxazines this derivative has the highest fluorescence efficiency (~ 0.5 in ethanol). Its fluorescence properties are closely related to those of Rhodamine 6G-ClO_4_. Absorption and fluorescence are shifted by about 100 nm to the red (fig. 7). As in the case of Oxazine 4, the fluorescence efficiency increases nearly a factor of 2 on deuteration of the amino groups. Apart from this effect, it is almost independent of solvent and temperature. However, variations of temperature have some effect on the absorption spectrum due to the annellated benzo group. In this respect Oxazine 4-ClO_4_ would be preferable. As pointed out earlier, these dyes cannot be used in basic solution. Being perchlorates, they are practically insoluble in water.

### Hexamethylindodicarbocyanine (HIDC) and Hexamethylindotricarbocyanine (HITC)

Very few dyes are known that are relatively stable and fluoresce well in the infrared region of the spectrum. Of the commercially available materials, the cyanine dyes 1,1′,3,3,3′,3′-hexamethylindodicarbocyanine (HIDC) iodide and 1,1′,3,3,3′,3′-hexamethylindotricarbocyanine (HITC) iodide stand out on both counts. Contrary to the other compounds

**Figure:**
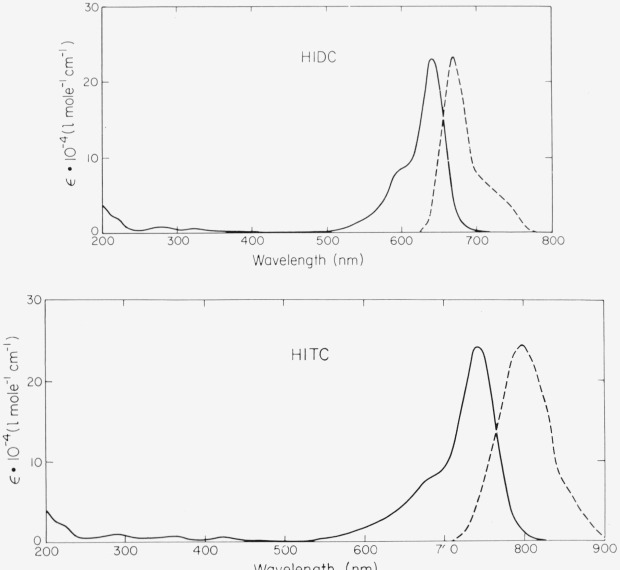


suggested here, the fluorescence efficiency of these dyes depends markedly on type of solvent and temperature. It appears to be highest in sulfoxides (dimethyl sulfoxide, tetramethylene sulfoxide). As shown in figure 8, the fluorescence of HITC extends to 900 nm. The photochemical stability is much higher than in related thiacarbocyanines. A concentrated solution of HIDC-iodide in dimethyl sulfoxide is currently in use in our laboratory as a photon counter that operates up to 700 nm. It is much more sensitive than equally concentrated solutions of methylene blue as this has a lower fluorescence efficiency.

The fluorescence spectra and efficiencies of the suggested dyes need to be determined accurately. To us this seems to be of greater benefit than perpetuating bad standards whose only justification today is their common usage.

## Figures and Tables

**Figure 8a, and b:**
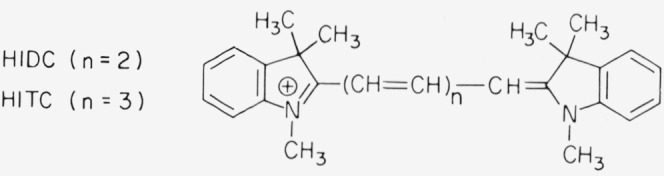
HIDC and HITC in ethanol. Absorption spectrum (ϵ molar decadic extinction coefficient); quantum spectrum of fluorescence (arbitrary units).

## Footnotes

1 Figures in brackets indicate the literature references at the end of this paper.

2 In order to describe materials and experimental procedures adequately, it is occasionally necessary to identify commercial products by manufacturer’s name or label. In no instance does such identification imply endorsement by the National Bureau of Standards, nor does it imply that the particular product or equipment is necessarily the best available for that purpose.
